# Determinants of late presentation to HIV/AIDS care in Southern Tigray Zone, Northern Ethiopia: an institution based case–control study

**DOI:** 10.1186/s12981-015-0079-2

**Published:** 2015-12-02

**Authors:** Yalemzewod Assefa Gelaw, Goitom Halefom Senbete, Akelew Awoke Adane, Kefyalew Addis Alene

**Affiliations:** College of Medicine and Health Sciences, Wollo University, Dessie, Ethiopia; Department of Epidemiology and Biostatistics, College of Medicine and Health Sciences, University of Gondar, Gondar, Ethiopia

**Keywords:** Late presentation, HIV/AIDS, Determinants, Case–control, Ethiopia

## Abstract

**Background:**

Late diagnosis and presentation to human immune deficiency virus (HIV)/acquired immune deficiency syndrome care reduce the benefits of antiretroviral therapy and increase the risk of HIV transmission.

**Objectives:**

This study was conducted to identify determinants of late presentation to HIV care among people living with HIV in Southern Tigray, Northern Ethiopia.

**Methods:**

An institution based un-matched case–control (1:2 ratios) supported with qualitative data was conducted in Southern Tigray Zone from March 1 to April 30, 2014. Individuals with HIV enrolled from six randomly selected health facilities were included in the study. Cases were people living with HIV who had cluster of differentiation four count <350 cells/μl or World Health Organization stages 3 or 4. A total of 442 study participants were included by systematic sampling techniques. Bivariable and multivariable binary logistic regression model was used to identify associated factors. Odds ratio with 95 % CI was computed to assess the strength of the associations.

**Result:**

Age categories, 25–29 years [AOR 3, 95 % CI (1.2–8.1)] and 35–39 years [AOR 4.1, 95 % CI (1.4–12.5)], having two [AOR 6, 95 % CI (1.3–28)] and more [AOR 5.2, 95 % CI (1.1–24.8)] lifetime sexual partners, poor social support [AOR 2.3, 95 % CI (1.26–4.30)], second (next to lowest) wealth quintile [AOR 3.3, 95 % CI 91.3–8.5)], fear of stigma [AOR 4.4, 95 % CI (2.2–8.3)], fear of losing job [AOR 6.8, 95 % CI (1.8–24.5)], and reported severe illness [AOR 4.3, 95 % CI (2.26–8)] were identified to be the risk factors for late presentation.

**Conclusion:**

Low socio-economic status and social support, fear of stigma were potential risk factors for late presentation. Efforts towards promoting early care seeking should target on these factors in the study area and other similar settings.

## Background

An estimated of more than 35.3 million adults were living with human immune deficiency virus (HIV) worldwide; and nearly two-third (69 %) of this were found in Sub-Saharan Africa [1]. The European Late Presenter Consensus Working Group (ELPCWG), defines “late presentation” as initial presentation to HIV care with a CD4 cell count of <350 cells/μl, and defines “presentation with advanced HIV disease” as presentation for care with a CD4 count below 200 cells/μl or any acquired immune deficiency syndrome (AIDS)-defining illness or event [2]. Clinically, late presentation limits opportunities to reduce further transmission [1, 2].

Introduction of antiretroviral therapy (ART) has substantially reduced the burden of HIV by decreasing the risk of death and improving treatment response. Late presentation for HIV care is associated with high risk of death, acquiring opportunistic infection and poor response to treatment [3–5]. Although people on ART have increased risk for non–AIDS illness, early initiations of ART is the option in developing countries including Ethiopia for people with HIV [6].

Previous studies and reports in Sub-Saharan Africa showed that 35–65 % of HIV patients initially present with a CD4 cell counts less than 200 cells/μl or with an AIDS-defining illness [6–9] and 20–40 % start ART with a CD4 counts <100 cells/μl. Such late presentation is associated with lower survival, early mortality and invasive bacterial and fungal infections [4]. It is also a major reason for the decreasing of retention rates in the course of ART [3, 5, 6, 10, 11]. In addition to it, late presentation also poses a higher cumulative risk of HIV transmission to others [6, 12, 13] as well as an economic burden to individuals particularly in the first few months of presentation [12].

Although findings on late presentation are limited, the problem of late ART initiation and its poor outcomes has been reported by some researches in Ethiopia [14–16].

Different scholars from developed [5, 6, 12] and developing countries [3, 5, 6] state various definitions, reasons and determinants of late presentation to HIV care. Studies from Ethiopia are scarce at diversified contextual/structural, clinical and individual level factors. Identifying the factors associated with late presentation for HIV care seeking is important for the improvement of HIV/AIDS prevention and control programs. It also helps to early undertakes for decisions makers and program managers on the upcoming revision of country wide HIV testing and care strategies. Therefore, it is important to identify the determinants of late presentation to HIV care.

## Methods

### Study design and study population

Un-matched case control study design supported with qualitative data was conducted from March 1 to April 20, 2014 (data collection period) in Southern Tigray Zone. The zone is located in the northern part of Ethiopia 645 km away from Addis Ababa, the capital city of Ethiopia. During the study period a total of 20 health facilities (3 general hospitals and 17 health centers) were providing voluntary counseling and testing, prevention of mother to child transmission, ART and treatment of opportunistic infection services.

The study population consisted of people over 18 years of age with HIV who were enrolled in HIV chronic care clinics and had visited the health facilities during the data collection period. Cases were people living with HIV/AIDS who had CD4 count less than 350 cell/mm^3^ or World Health Organization (WHO) clinical stage III or IV during their first clinical visit for HIV care. Controls were PLWHA whose CD4 count was ≥350 cell/μl and WHO clinical stage I or II. Focus group discussions (FGD) were conducted with PLWHA who were a member of mother’s supporting group.

### Sampling techniques and sample size determination

The sample size was calculated using Epi Info version 7.0 by considering the following assumptions: the proportion of HIV status non-disclosure to partners among the cases was 46.2 and 29.4 % among the controls [17], using 95 % CI, 90 % power and case to control ratio of 1 to 2 to detect an odds ratio of 2. Then by adding 10 % non-response rate, the final sample size was 442 (147 cases and 295 controls). Previous studies have indicated that non disclosure of HIV status to partners, alcohol use and perception of stigma increase for late presentation. Non disclosure of HIV status to partner was selected to maximize sample size.

Simple random sampling techniques were applied to select respondents. Two district hospitals and four health centers were selected randomly.

After collecting baseline CD4 count and/or WHO staging using chart reviews, people living with HIV/AIDS were classified in two groups before data collection began. Cases and controls were then selected using systematic random sampling techniques based on visits that occurred during the data collection period. We divided the sample among the six participating health institutions with a ratio of five cases to two controls. HIV-positive individual’s under 18 years of age, those with neither CD4 count data nor WHO HIV/AIDS stage recorded at first visit, and those who were overtly cognitively impaired were excluded from the study.

### Data collection and analysis

The data were collected using an interviewer administered technique by 11 trained clinical nurses working in ART clinics and supervised by two health officers. Pre-coded and structured questionnaires consisted of socio-demographic, socioeconomic, behavioral, psychosocial and social environments were used. Data collectors and supervisors were trained with respect to the objectives of the study; confidentiality of information, respondent’s rights, and interviewing technique prior to the actual data collection.

Wealth quintile information was by asking about the presence or absence of durable assets and services at the house hold level. Different indicators were used for urban and rural areas to measure wealth quintiles. The index was constructed for each household via principal components analysis. Indicators having ≥50 % rescaled communalities were used to construct wealth index and further categorized into five wealth quintiles (as lowest, second, third, fourth and highest based on the Ethiopian demographic and health survey classification [18].

Data were entered to Epi Info version 7 and transferred to SPSS 20 for further statistical analysis. Descriptive and summary statistics were computed. Both bivariable and multivariable logistic regression models were used to identify associated factors. Variables having p value less than 0.2 in the bivariable analysis were remained in the multivariable model to control the effect of confounders. The Hosmer–Lemeshow goodness-of-fit statistic was used to assess the fitness of the model. Odds ratios (OR) with their 95 % confidence intervals (95 % CI) were calculated to measure the strength of association. P value less than 0.05 were considered to be significant determinants for cases and controls.

Qualitative data were collected by the principal investigator through FGD parallel to quantitative data collection. The recorded data were transcribed, translated and analyzed using thematic analysis under the sub headings of the quantitative findings and new themes for new findings.

### Ethical consideration

This study was carried out after receiving ethical approval from the Institutional Review Committee of University of Gondar. Prior to approval, the proposal was provided to reviewers to assure the ethical issues. Permission letter was also obtained from the respective health institutions (medical director or persons in charge of institutions) to identify cases and controls through chart review by authorized ART case managers or ART nurses. Before interviewing or recording, the data collector fully explained the purpose of the study to each participant and obtained written informed consent from each participant. To ensure confidentiality, names were not mentioned in the questionnaire and result report. In addition, the collected data were locked.

## Result

A total of 413 HIV positive individuals (147 cases and 266 controls) participated in this study. The median age (IQR) at time of presentation was 30 (±13) years. The median age (IQR) at time of presentation for cases and controls were 30 (±11) and 30 (±13), respectively (Table 1).

**Table 1:** Socio-demographic characteristics at first presentation of cases and controls, Southern Tigray zone, Northern Ethiopia, 2014

Variables	Categories	Cases, N (%)	Controls, N (%)
Sex	Male	55 (37.4)	86 (32.3)
	Female	92 (62.6)	180 (67.7)
Marital status at first presentation	Married	73 (49.7)	122 (45.9)
	Widowed	22 (14.9)	37 (13.9)
	Divorced	34 (23.1)	67 (25.2)
	Never married	18 (12.2)	40 (15)
Pregnancy (18–49)	Yes	11 (12.4)	29 (17)
	No	78 (87.6)	141 (82.9)
Education at first presentation	Can’t read and write	87 (59.2)	167 (62.8)
	Primary (1–8)	40 (27.2)	74 (27.8)
	Secondary and above	20 (13.6)	25 (9.4)
Residence	Urban	93 (63.3)	154 (57.9)
	Rural	54 (36.7)	112 (42.1)
Occupation	Farmer	34 (23)	79 (29.7)
	Professional/business occ.	37 (25)	49 (18.4)
	Unemployed	25 (17)	45 (16.9)
	House wife	27 (18.4)	53 (19.9)
	Daily laborer	24 (16.3)	40 (15)
Age category	18–24	19 (12.9)	63 (23.7)
	25–29	35 (23.8)	48 (18)
	30–34	36 (24.5)	58 (21.8)
	35–39	28 (19)	32 (12)
	40–44	16 (10.88)	32 (12)
	45+	13 (8.8)	33 (12.4)
Wealth quintiles (SES)	Lowest	40 (27.2)	42 (15.8)
	Second	39 (20.4)	47 (17.7)
	Third	24 (16.3)	57 (21.4)
	Fourth	26 (17.7)	59 (22.2)
	Highest	18 (12.2)	61 (22.9)

### Determinants of late presentation

Age group, having had multiple sexual partners, poor socioeconomic status, non-disclosure status to partner, presentation before 2011, low household social support, desire to protect family, severity of illness, fear of stigma and fear of losing job were all found to be independent risk factors for late presentation to HIV/AIDS care (Table 2).

**Table 2:** Binary and multiple logistic regressions showing determinants of late presentation to HIV/AIDS care, among PLWHA receiving HIV care, Southern Tigray zone, Northern Ethiopia, 2014

Determinants/factors	Presentation to HIV care		COR (95 % CI)	AOR (95 % CI)
	Cases (%)	Control (%)		
Year of presentation				
Before 2008	37 (25.2)	46 (17.3)	1	1
2009–2010	62 (42.2)	65 (24.4)	1.2 (0.681–2.06)	0.76 (0.33–1.73)
2011–2012	27 (18.4)	65 (24.4)	0.51 (0.277–0.963)	0.31 (0.12–0.77)**
2013–2014	21 (14.3)	90 (33.8)	0.29 (0.153–0.552)	0.14 (0.05–0.37)**
Age category				
18–24	19 (12.9)	63 (23.7)	1	1
25–29	35 (23.8)	48 (18)	2.4 (1.23–4.73)	3.0 (1.15–8.12)*
30–34	36 (24.5)	58 (21.8)	2.0 (1.06–3.98)	1.4 (0.52–3.65)
35–39	28 (19)	32 (12)	2.9 (1.41–5.96)	4.1 (1.35–12.46)*
40–44	16 (10.88)	32 (12)	1.7 (0.75–3.65)	1.1 (0.34–3.71)
45+	13 (8.8)	33 (12.4)	1.3 (0.62–3.29)	1.3 (0.37–4.23)
No. of life time sexual partners				
None	4 (2.7)	28 (10.5)	1	1
One	49 (33.3)	77 (28.9)	4.4 (1.47–13.47)	4.0 (0.86–18.59)
Two	46 (31.3)	98 (36.8)	3.3 (1.08–9.9)	6.0 (1.28–28.02)*
More than two	48 (32.7)	61 (22.9)	5.5 (1.80–16.7)	5.2 (1.08–24.76)*
Presence of HIV infected HH member				
Yes	16 (10.9)	51 (34.7)	1	
No	131 (89.1)	215 (65.3)	1.9 (1.06–3.54)	
Presence of HIV care attendee				
Yes	14 (9.5)	44 (16.5)	1	
No	133 (90.5)	222 (83.5)	1.8 (1.1–3.56)	
Witnessed stigma				
Low stigma	91 (61.9)	184 (69.2)		
High stigma	56 (38.1)	82 (30.8)	1.4 (0.90–2.10)	
Depression				
Not depressed	56 (38.1)	154 (57.9)	1	
Depressed	91 (61.9)	112 (42.1)	2.2 (1.47–3.37)	
Level of social support				
Poor social support	103 (70)	95 (35.7)	4.2 (2.73–6.49)	2.3 (1.26–4.30)*
Good social support	44 (30)	171 (64.3)	1	1
Wealth quintile (SES)				
Lowest	40 (27.2)	42 (15.8)	3.2 (1.63–6.37)	1.05 (0.38–2.83)
Second	39 (20.4)	47 (17.7)	2.8 (1.43–5.52)	3.3 (1.31–8.46)*
Third	24 (16.3)	57 (21.4)	1.4 (0.70–2.90)	0.8 (0.27–2.31)
Fourth	26 (17.7)	59 (22.2)	1.5 (0.74–3.0)	1.1 (0.44–2.82)
Highest	18 (12.2)	61 (22.9)	1	1
Disclosure to partner				
Yes	26 (35.6)	72 (59)	1	1
No	47 (64.4)	50 (41)	2.6 (1.43–4.74)	2.0 (1.05–4.14)*
HIV support groups in community				
Yes	70 (47.6)	150 (56.4)	0.70 (0.47–1.05)	
No	77 (52.4)	116 (43.6)	1	
Diet and life style change for positive result				
Yes	49 (33.3)	122 (45.9)	0.59 (0.39–0.89)	0.45 (0.24–0.87)*
No	98 (66.6)	144 (54.1)	1	1
Desire to protect family				
Yes	23 (15.6)	126 (43.4)	0.20 (0.12–0.34)	0.19 (0.09–0.37)**
No	124 (84.4)	140 (52.6)	1	1
To know CD4 count				
Yes	5 (3.4)	33 (12.4)	0.25 (0.095–0.65)	0.37 (0.09–1.43)
No	142 (96.6)	233 (87.6)	1	1
Severity of illness				
Yes	89 (60.5)	66 (24.8)	4.7 (3.01–7.16)	4.3 (2.26–8.0)**
No	58 (39.5)	200 (75.2)	1	1
Fear of stigma				
Yes	94 (63)	65 (24.5)	5.5 (3.5–8.5)	4.4 (2.2–8.3)**
No	53 (37)	201 (75.5)	1	1
Fear of disclosure				
Yes	60 (40.8)	40 (15)	3.9 (2.43–6.23)	2.0 (0.99–4.32)
No	87 (59.2)	226 (84.9)	1	1
Fear of losing job/income source				
Yes	27 (18.4)	6(2.3)	9.8 (3.92–24.2)	6.8 (1.8–24.54)*
No	120 (81.6)	260 (97.7)	1	1

* Significant (p ≤ 0.05)

** Highly significantly (p < 0.001)

Participants in the age group of 25–29 years [AOR 3, 95 % CI (1.15–8.12)] and those 35–39 years [AOR 4.1, 95 % CI (0.35–12.46)] were 3 and 4.1 times more likely to present late, respectively, as compared to those 18–24 years of age.

Year of presentation was associated with likelihood of late presentation. Those study participates who were visiting the HIV care before 2008 were presenting lately. The risk of late presentation to HIV care among PLWHA who presented in the years 2011–2012 [AOR 0.31, 95 % CI (0.12–0.77)] and 2013–2014 [AOR 0.14, 95 % CI (0.05–0.37)] was decreased by 69 and 86 %, respectively, as compared to those presenting before the year 2008.

Presence of more than one lifetime sexual partner was also associated with late presentation to HIV care. HIV-positive individuals with two lifetime sexual partners were six times [AOR 6, 95 % CI (1.28–28.02)] more likely to present late as compared with participants with none. Those with more than two life time sexual partners were also 5.2 times [AOR 5.2, 95 % CI (1.08–24.76)] more likely to present late compared to those with none.

The second (next to lowest) wealth quintile of HIV-positive individuals was 3.3 times [AOR 3.3, 95 % CI (1.31–8.46)] more likely to present late as compared to the highest wealth quintile group.

Partner or spousal HIV status disclosure was found to deter presentation. Married participants who did not disclose their HIV status to their partner were two times more likely to present late [AOR 2, 95 % CI (1.05–4.14)] compared to those who did.

HIV positive individuals who had poor house hold social support were 2.3 times more likely to present late as compared to those who had good social support at home [AOR 2.3, 95 % CI (1.26–4.30)].

Barriers perceived by PLWHA were also associated with late presentation. Participants who reported fear of stigma were 4.4 times more likely to present late as compared to those who did not [AOR 4.4, 95 % CI (2.23–8.27)]. Participants who reported fear of losing their source of income were 6.8 more likely to present late as compared to those who did not [AOR 6.8, 95 % CI (1.8–24.54)].

The way participants perceived the benefits of HIV care also contributed to discrepancy in stage at presentation. Participants who responded that “protecting family” was their reason for initiating care were 81 % less likely to present late, as compared to those who did not [AOR 0.19, 95 % CI (0.09–0.37)]. By contrast, participants who responded that “severe illness” was a reason for initiating care were 4.3 times more likely to present late as compared to those who did not [AOR 4.3, 95 % CI (2.26–8.0)].

The qualitative results revealed that fear of stigma, fear of losing income source/former job, use of alternative therapies, lack of familial pressure to initiate care, inadequacy of social support (spouse, friends and religious leaders) and inadequate knowledge about delay to care were the barriers reported by people living with HIV/AIDS in the study area (Table 3).

**Table 3:** FGD results extracted from HIV positive individuals works on mothers supporting groups concerning barriers to early HIV/AIDS care, Southern Tigray Zone, April 2014

Barriers to HIV/AIDS care	HIV/AIDS positive individuals working on Mother Supporting Group
Fear of stigma	“… I was worried that people will talk about me if they hear I tested positive”… Male-32 “When I became aware of my HIV status my challenge was fear of health professionals and seeing other individuals I know while attending the ART clinic”, Female-27.
Fear of losing income source/former job	“People seem supportive towards HIV positives at first, but the pain is that they gradually change their feeling and treat you as ‘too weak’ to accomplish your duty …HIV positives will be shifted to lower status/position”, Male-55 “Although they are HIV positive, numerous commercial sex workers in the town continue their commercial sex work”, Female-27
Use of non-medical treatment	“…then she ate a dried Wild boar’s meat and Wild boar’s bile”, Female-30 “…he told me a very bitter plant called ‘Oure’ or ‘Eret’ is medicine for HIV and he had been drinking it until he ended up dying”, Female-30 “I went to a local traditional healer called ‘TonqualIi’ or magician and spent around 3000 Ethiopian Birr then he told to my wife wash my body using sheep’s blood and dung added together ….”, Male-38 “There was one HIV-positive neighbor with symptoms of diarrhea, she bought drugs from the pharmacy, was relieved of her symptoms for some time and then went to other pharmacy a few days later….”, Female-30 “My husband and were severely diseased and confined to bed for around 6 months. He had been telling that his illness was due to other diseases like malaria, and he has been getting medicines from drug stores”, Female-34
Low families driving force for initiation care	“We should let peoples think for the next generation…If my children are healthy…..”, Male -32 … “My child is negative”, Female-27, “My wife and children are negative”, Males-32, 55
Inadequacy of social support (spouse, friends and religious leaders)	“… I show him my ART drugs that I am taking and he became persuaded to take his treatment too”, Female-55 “I become angry and decided not to take any treatment for 1 year. Later on my friends were taking HIV treatment and they encouraged me to take treatment.” Male-28 “Males say that we are healthy even though we have the HIV and nothing bad is going on us, but, females become ill and suffer from headache and they come to ART clinics saying I tested positive for HIV …”, Female-27 “….later on my wife went to the health center and tested positive after that she encouraged me to be tested …”, Male-38 “…..I then persuaded my husband to be tested for HIV and avoided taking other drugs from outside the treatment center’’, Female-34 “My brother shouts at me, you should be tested and know the cause for your illness today; I will help every expense that follows …..”, Female-35 “…she was severely ill and her family said that they didn’t want to take care of the dead”, Female-27
Inadequate knowledge	“The HIV testing sites that are conducted in the shade in town are always crowded with people being tested, especially males. But they don’t come to care once they have tested positive [F-55] and they can only be accessed after they become diseased, arriving on a stretcher or by ambulance, or when found during house-to-house tracing by volunteer HIV support groups. …”, Female-34

## Discussion

This study identified determinants of late presentation to HIV/AIDS care in ART services are accessible at primary health care units. Although different studies reported various risk factors for late presentation, the present study assessed people’s perceived need for care, perceived barriers to HIV care, socioeconomic status, and the role of social support.

Consistent with previous studies in Europe and Africa [11, 19–22, 29] HIV-positive participants were present late for care when their age gets old. It has been posited that when age increase adults are more likely to have a moral or ethical reason for presenting earlier to care. However, a study in India reported young adults present late [23]. This could be explained by differences in method and project design, as well as varying social and cultural issues. In countries like Ethiopia, old peoples considered as immaculate. In the context of the study setting elder individuals are a pronounced in the milieu.

Participants with a history of multiple sexual partners were more likely to present late to care. This was also reported in previous study in Ethiopia [17]. However, in Uganda [11] and Venezuela [24] HIV-positive individuals with more than one sexual partner presented early. One factor for this different finding might be the emphasis of taboo on sexually active behaviors in Ethiopian society, and an increased propensity to feel embarrassment regarding this behavior [25]. Although having legal multiple sexual partners are practiced in Islamic religions of Ethiopia, seeking treatment related to sexual transmitted disease is still lack.

Socioeconomic positions of the study participants were also the determinant factors to present late for HIV care. Participants in the second (next to the lowest) wealth quintile were statistically associated with late presentation. This was also consistent with previous systematic review of three indicators of socio economic status (education, income and occupation) [26]. Despite the difference in measurement of socioeconomic positions of HIV positive individuals, some studies also describe income as a determinant factor for late presentation [6, 14, 17]. In the context of Ethiopian health care delivery system voluntary counseling and testing, diagnosis and ART services are exempted.

In Ethiopia peoples are most of the time living together; shares the same dish and chalice in different occasions. Participants with poor social support were more likely present late, consistent with studies done in Durban, South Africa [14] and Houston, Texas [27]. Here we believe that social stigma and lack of social support can contribute towards people feeling inhibited in seeking HIV/AIDS care. The qualitative data also support this finding. Behavioral change and communication concerned with social stigma and poor social support needs to be done using key informants, religious person and mother supporting groups.

Poor partner’s communication on reproductive health matters has an impact for health status of the family. Partners who did not disclose their status to their partner were more likely to present late. Similarly, studies done in Uganda [6], Kenya [28] and Ethiopia [17], revealed that disclosure to partners can initiate timely HIV/AIDS care. HIV/AIDS related stigma and discrimination has declined dramatically in Ethiopia and support following disclosure has improved.

The current study reported that participants who had been visiting health facilities in recent years were more likely to present early as compared to those visited health facilities before 2008. This finding is consistent with studies done in Nigeria [29], Belgium and France [22], New York City [30] and India [23]. This could be due to the increased access to care, overall awareness of HIV, media accessibility and a reduction in stigma over time [31–33].

Fear of stigma and losing one’s job were determinant factors for late presentation consistent with previous studies in Sub-Saharan Africa [34] and Zimbabwe [35]. This is also supported by the qualitative FGD report that showed fear of stigma and losing job/customers were barriers. This is could be common problem of female participant’s sex worker.

Our study also showed that participants who perceived HIV as a sever disease were more likely to seek care early. This is demonstrated in the qualitative data in which protecting family members was a driver to initiate HIV care early.

In this study the use of traditional medicine and a holy water as a treatment and sex of respondents were not statistically significant with late presentation to HIV/AIDS care. However, data from the FGD indicates that male’s patients who had visited traditional medicine and/or holy water sites were more likely to present late for HIV/AIDS care. This discrepancy could be due to that HIV positive individuals might fear to disclose the utilization of these services. It could be also due to the rareness of such traditional treatments.

The potential limitations of this study were that the study may have been prone to recall biases and that there may have been misclassification of presentation or diseases do to social pressure.

## Conclusion

Age older than 25, having multiple sexual partners, poor socioeconomic status, non-disclosure to partner, presentation before 2011 for care, low house hold social support, desire to protect family, severity of illness, fear of stigma and fear of losing job were all found to be independent risk factors for late presentation to HIV/AIDS care.
